# *Trans*-Fatty Acids Aggravate Obesity, Insulin Resistance and Hepatic Steatosis in C57BL/6 Mice, Possibly by Suppressing the IRS1 Dependent Pathway

**DOI:** 10.3390/molecules21060705

**Published:** 2016-05-30

**Authors:** Xiaona Zhao, Cheng Shen, Hong Zhu, Cong Wang, Xiangwei Liu, Xiaolei Sun, Shasha Han, Peng Wang, Zhen Dong, Xin Ma, Kai Hu, Aijun Sun, Junbo Ge

**Affiliations:** 1 Shanghai Institute of Cardiovascular Diseases, Zhongshan Hospital, Fudan University, 180 Feng Lin Road, Shanghai 200032, China; zhaoxiaona1224@sina.cn (X.Z.); shencheng1117@126.com (C.S.); zhuhongisme@126.com (H.Z.); 13211210017@fudan.edu.cn (C.W.); Liuxiangwei1224@126.com (X.L.); 18516089416@sina.cn (X.S.); zxc1990217@sina.com (S.H.); 18463739133@163.com (P.W.); Fionazhao123@aliyun.com (Z.D.); Moveforward2013@yean.net (X.M.); hu.kai@zs-hospital.sh.cn (K.H.); 2 Institute of Biomedical Science, Fudan University, Shanghai 200032, China

**Keywords:** *trans*-fatty acid, obesity, insulin resistance, hepatic steatosis

## Abstract

*Trans*-fatty acid consumption has been reported as a risk factor for metabolic disorders and targeted organ damages. Nonetheless, little is known about the roles and mechanisms of *trans*-fatty acids in obesity, insulin resistance (IR) and hepatic steatosis. Adult C57BL/6 male mice were fed with four different diets for 20 weeks: normal diet (ND), high fat diet (HFD), low *trans*-fatty acids diet (LTD) and high *trans*-fatty acid diet (HTD). The diet-induced metabolic disorders were assessed by evaluating body weight, glucose tolerance test, hepatic steatosis and plasma lipid profiles post 20-week diet. Histological (H&E, Oil-Red-O) staining and western blot analysis were employed to assess liver steatosis and potential signaling pathways. After 20-weeks of diet, the body weights of the four groups were 29.61 ± 1.89 g (ND), 39.04 ± 4.27 g (HFD), 34.09 ± 2.62 g (LTD) and 43.78 ± 4.27 g (HTD) (*p* < 0.05), respectively. HFD intake significantly impaired glucose tolerance, which was impaired further in the mice consuming the HTD diet. The effect was further exacerbated by HTD diet. Moreover, the HTD group exhibited significantly more severe liver steatosis compared with HFD group possibly through regulating adipose triglyceride lipase. The group consuming the HTD also exhibited significantly reduced levels of IRS1, phosphor-PKC and phosphor-AKT. These results support our hypothesis that consumption of a diet high in *trans*-fatty acids induces higher rates of obesity, IR and hepatic steatosis in male C57BL/6 mice, possibly by suppressing the IRS1dependent pathway.

## 1. Introduction

*Trans*-fatty acids are a kind of unsaturated fatty acid that are uncommon in Nature, but commonly produced industrially from vegetable fats for use in margarines, snack foods, packaged baked goods and fried foods starting in the 1950s [1,2]. According to epidemiological investigations, *trans*-fatty acid consumption was associated with increased risk of cardiovascular disease and metabolic disorders [3]. In 2015, the Food and Drug Administration (FDA) of USA declared that *trans*-fatty acids should not be added into manufactured food without special permission. However, many other countries such as Mexico still have high consumption of *trans*-fatty acids, while countries in Asian like China have only proposed to restrict the consumption of *trans*-fatty acid without an enforcement policy [4].

Accumulating evidence suggests that *trans*-fatty acids are involved in obesity and insulin resistance (IR) [5], but the underlying mechanism is not fully understood. Recent studies have reported that high fat diet and high *trans*-fatty acid levels could induce nonalcoholic fatty liver disease by oxidative stress [6]. Nonalcoholic liver steatosis could progress to steatohepatitis, hepatic fibrosis and even worse [7]. Up to now, the direct evidence and studies on the molecular mechanism of action of *trans*-fatty acids in the lipid disorders, including liver steatosis are insufficient. Previous studies showed that diacyglycerol acyl synthetase (DGAT1), lipoprotein lipase (LPL), adipose triglyceride lipase (ATGL) and acyl-CoA synthetase (ACSL1) were involved in the processes of fatty acid transfer and uptake and lipid turnover [8,9,10,11]. Changes in those critical enzymes caused by over consumption of fatty acids were shown to be linked with excessive fat deposition in the liver [12]. Glenn *et al.* demonstrated that *trans*-fatty acids could enhance lipid accumulation in the heart by modulating DGAT1 [13]. Besides, several signaling pathways were shown to be closely related with IR, such as the classical IRS1-PI3K-Akt pathway, JNK-dependent pathway and IL6-STAT3 signaling pathways [14]. These studies suggested *trans*-fatty acids could play a vital role on hepatic steatosis and IR.

The purpose of this study was to investigate the effects and underlying mechanisms of *trans*- fatty acid in obesity, IR and hepatic steatosis. Our hypothesis was that *trans*-fatty acids might induce more severe obesity, IR and hepatic steatosis in comparison with a high fat diet through regulating DGAT1, ATGL and ACSL1, and IRS1 dependent pathways.

## 2. Results

### 2.1. High Fat and Trans-Fatty Acid Diet Induces Obesity and Dyslipidemia

**Figure 1:**
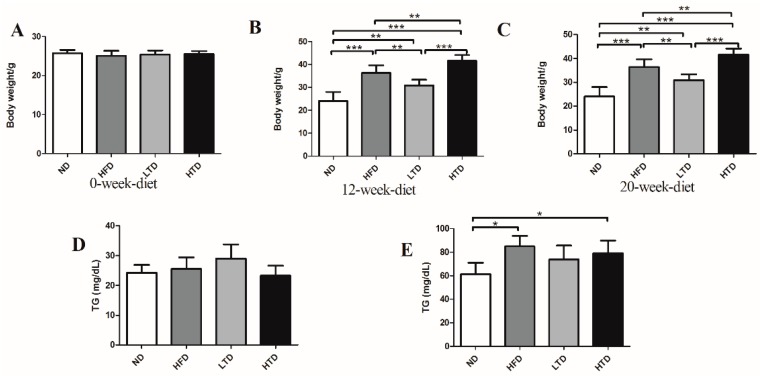

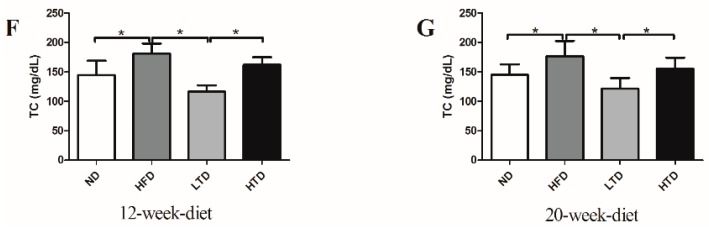
Body weight change and lipid profile at 12 and 20 weeks among various group. (**A**–**C**) Body weight of ND, HFD, TD and HTD group at 0 week, 12 weeks and 20 weeks; (**D**–**E**) TG at 12 weeks and 20 weeks; (**F**–**G**) TC of the four groups at 12 weeks and 20 weeks. (*n* = 8 per group). *: *p* < 0.05, **: *p* < 0.01, ***: *p* < 0.001, Results are shown as mean ± SD.

In order to observe the effects of HFD and the *trans*-fatty acid diet on obesity, the body weight of mice was measured in the different groups of animals at 0 week, 12 weeks and 20 weeks. As shown in Figure 1A–C, though the calories were similarly low in the ND and LTD groups, the body weight was significantly higher in the LTD group than in the ND group at 12 and 20 weeks, as expected, body weight was significantly higher in HFD and HTD groups than in ND and LTD groups at 12 and 20 weeks. This suggests that *trans*-fatty acid can induce obesity. Serum TG level was also significantly increased in HFD and HTD groups compared to ND group, and TC level was higher in HTD group than LTD group at 12 and 20 weeks, but both parameters were similar between LTD and ND, as well as between HFD and HTD groups after 12 and 20 weeks of diet. (Figure 1D–G).

### 2.2. Fat Mass and White Adipose Tissue

**Figure 2:**
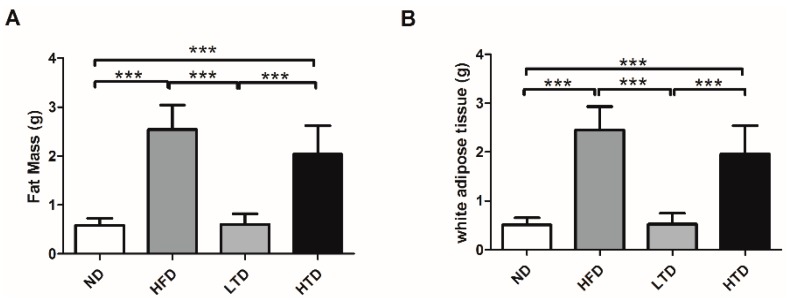
The fat mass and sections of white adipose tissue after 20 weeks diet. (**A**) The fat mass of ND, HFD, TD and HTD group at 20 weeks; (**B**) Sections of white adipose tissue of ND, HFD, TD and HTD group at 20 weeks. ***: *p* < 0.001, Results are shown as mean ± SD.

In our study, we tested the fat mass and sections of white adipose tissue after 20 weeks of diet. Compared with the ND and LTD groups, the fat mass and the sections of white adipose tissue were higher in the HFD and HTD groups, but there was no significant difference between the ND and LTD, HFD and HTD groups (Figure 2).

### 2.3. Impact of Trans Fatty Acid and High Fat Diet on Glucose Tolerance

**Figure 3:**
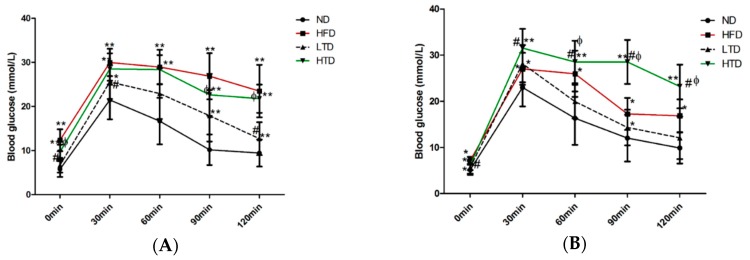
(**A**) Glucose tolerance test of various groups post 12 weeks diet after intraperitoneal injection of 2 g/kg glucose; (**B**) Glucose tolerance test of various groups post 20 weeks diet after intraperitoneal injection of 2 g/kg glucose. *n* = 6–8 per group. *: *p* < 0.05; **: *p* < 0.01 *vs.* ND group; #: *p* < 0.05 *vs.* HFD group; φ: *p* < 0.05 *vs.* LTD group. Results are shown as mean ± SD.

Previous studies demonstrated that obesity was associated with IR and T2DM [15]. In order to observe the role of *trans*-fatty acid and high fat diet on glucose metabolism, a glucose tolerance test was performed in mice after 12 and 20 weeks of feeding various diet regimens. As shown in Figure 3A,B, the blood glucose concentration was comparable between the ND and LTD groups at 0 and 60 min post intraperitoneal injection of glucose (2 g/kg), however, glucose levels at 30, 90 and 120 min were significantly higher in the LTD group than in the ND group at 12 weeks (Figure 3A), indicating that LTD could induce mild IR. Blood glucose concentration was significantly and equally increased at 0 to 120 min in both the HFD and HTD groups compared to the ND group after 12 weeks (Figure 3A), indicating that HFD and HTD induced significant IR at this time point. After 20 weeks, LTD still induced mild IR and both HFD and HTD induced significant IR (Figure 3B). It is noteworthy that glucose levels at 60, 90 and 120 min were significantly higher in the HTD group than in the HFD group, suggesting HTD induced more significant IR compared to HFD.

### 2.4. Impact of Trans Fatty Acid and High Fat Diet on Hepatic Steatosis

**Figure 4:**
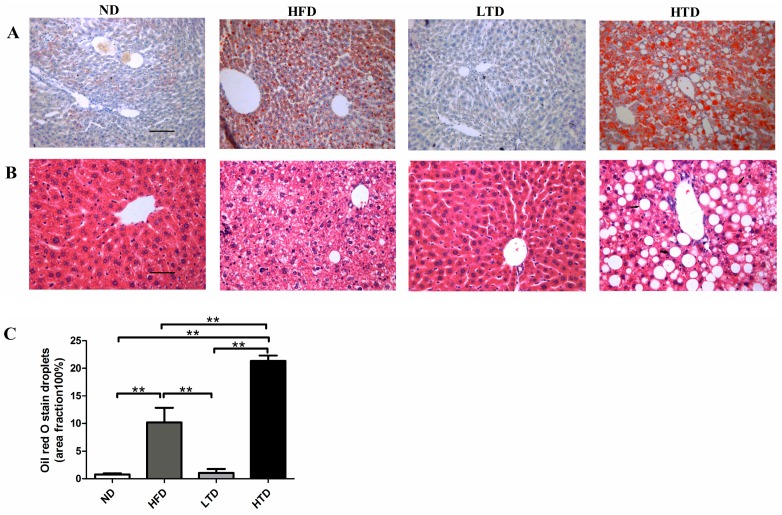
Hepatic histology. (**A**) Oil-Red-O staining of liver sections in ND, HFD, TD and HTD groups post 20 weeks diet. Micrograph exhibiting oil droplets levels in the liver (magnification: 200× *g*) and red droplets in the picture exhibiting lipid droplets; (**B**) H&E staining of liver sections in ND, HFD, TD and HTD groups post 20 weeks diet (magnification: 200×) and white vacuoles representing lipid droplets. Scar bars indicate 100 μm; (**C**) Quantitative analysis of oil droplets, **: *p* < 0.001. Data are shown as mean ± SD.

Recent studies have indicated that IR was associated with liver steatosis and both IR and hepatic steatosis belong to metabolic disorder [16]. H&E and ORO staining were therefore performed on liver samples. Normal morphology of hepatocytes was observed in the ND and LTD groups (Figure 4A,B). No neutral lipid accumulations were seen in the ND and LTD groups (Figure 4A–C). However, significant neutral lipid disposition was evidenced in the HFD and HTD groups. Moreover, the lipid droplets area fraction was much more in the HTD group compared with the HFD group (Figure 4A–C). Collectively, these data demonstrated that high *trans*-fatty acid induced more severe hepatic steatosis than HFD.

### 2.5. Hepatic Expression of DGAT1, ATGL and ACSL1 of Various Groups

**Figure 5:**
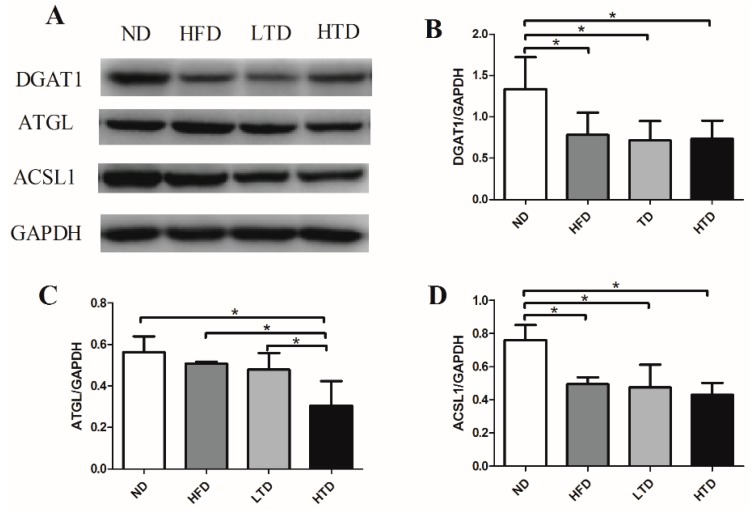
Effect of *trans*-fatty acids on lipid-relevant proteins. (**A**) Representative gel blots depicting lipid-relevant proteins from liver tissue after 20 weeks of diet, including DGAT1, ATGL and ACSL1 (GAPDH used as loading control) (*n* = 5–6 per group); (**B**) DGAT1 expression; (**C**) ATGL expression; (**D**) ACSL1 expression. Data were shown as mean ± SD, *: *p* < 0.05.

As shown in Figure 5A–D, compared with the ND group, DGAT1 was significantly decreased in liver of the HFD, LTD and HTD groups. Decreased ATGL was found in the HTD group and decreased ACSL1 was evidenced in the HFD, LTD and HTD groups. The above results suggested that lipid turnover was suppressed in the HFD, LTD and HTD groups. Accordingly, hepatic steatosis was the worst in the HTD group post 20-week-diet. These results might suggest that decreased ATGL induced by a high trans-fatty acid diet might be linked with the more severe hepatic steatosis evidenced in the HTD group.

### 2.6. Impact of Trans-Fatty Acid and High Fat Diet on the Insulin Resistance Signaling Pathway

**Figure 6:**
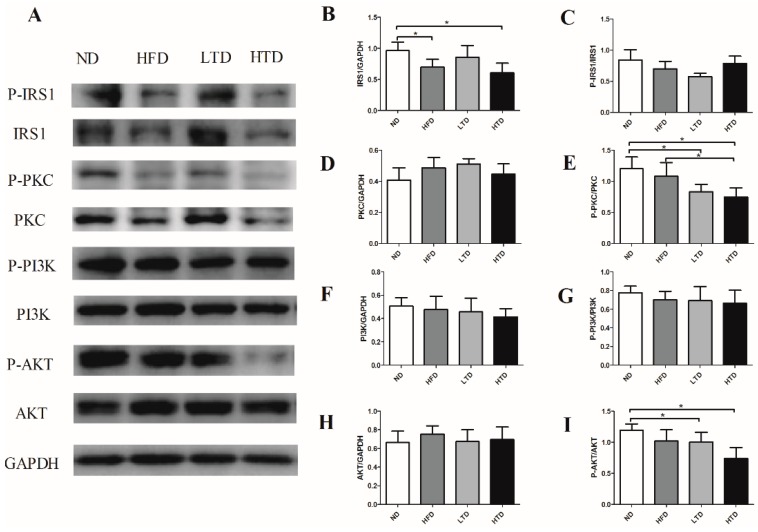
Effects of *trans*-fatty acid on the insulin signal pathway. (**A**) Representative gel blots depicting proteins in the insulin signal pathway of total and phosphorylated insulin receptor substrate1 (IRS1), Protein Kinase C (PKC), Phosphatidylinositol 3-kinase (PI3K) and AKT from liver tissue after 20 weeks diet (GAPDH used as loading control) (*n* = 4–6 per group); (**B**) Total IRS1 expression (**C**) IRS1 phosphorylation (Ser302, P-IRS1 to total IRS1 ratio); (**D**) Total PKC expression; (**E**) PKC phosphorylation (Ser359, P-PKC to total PKC ratio); (**F**) Total PI3K expression; (**G**) PI3K phosphorylation (p85 (Tyr458)/p55 (Tyr199), P-PI3K to total PI3K ratio); (**H**) Total AKT expression; (**I**) AKT phosphorylation (Ser473, P-AKT to total AKT ratio). Data were shown as mean ± SD, *: *p* < 0.05.

To further elucidate a potential signaling pathway involved in *trans*-fatty acid diet and high fat diet-induced IR, the insulin receptor substrate 1 (IRS1) dependent pathway was examined in liver samples. Our results showed that the total IRS1 expression was significantly decreased in the HFD and HTD groups, but the phosphorylated IRS1 was similar among groups (Figure 6A–C). It has been reported that PI3K was mainly expressed in muscle. In line with this fact, we did not observe significant differences of PI3K and phosphor-PI3K expression in liver tissues among the four groups (Figure 6A,D–E) [17]. PKC δ at Ser359 was significantly lower in the LTD and HTD groups compared to the ND group. AKT at Ser473 was lower in the LTD and HFD groups as compared with the ND group (Figure 6A,F–I). These results indicate that IR induced by *trans*-fatty acids might at least partly mediated by suppressing the expressions of IRS1, PKC and AKT.

## 3. Discussion

The major findings of our work are as follows: HTD induces more severe obesity, IR and hepatic steatosis compared to HFD in male C57BL/6 mice. Since obesity, IR and hepatic steatosis are the major metabolic disorder components, our results might indicate that excessive *trans*-fatty acid consumption might contribute to the development of metabolic syndrome. Moreover, we demonstrated that a *trans*-fatty acid diet downregulated ATGL expression and suppressed IRS-1dependent IR signaling in the hepatic tissue. These findings might serve as potential working mechanisms for *trans*-fatty acid-induced metabolic disorders.

Our finding in male C57BL/6 mice is in line with the epidemiological study results showing that *trans*-fatty acid were a risk factor of obesity and metabolism syndrome [18]. Notably, we demonstrated that glucose intolerance induced by high *trans*-fatty acid diet was more severe than that induced by a high fat diet. This finding is linked with more severe decrease on two IR-related signaling systems: phosphorylated PKC (Ser559, P-PKC to total PKC ratio) and phosphorylated AKT (Thr308, P-AKT to total AKT ratio), in hepatic tissue of HTD mice compared to HFD mice. Thus, high *trans*-fatty acid levels might induce more severe IR, possibly through downregulating hepatic phosphorylated PKC and phosphorylated AKT. Our results are thus in line with previous finding in that high fat diet for eight months induced insulin resistance via suppression of the phosphorylation of IRS1 [19]. However, our data demonstrated that only the total IRS1 was decreased in HFD and HTD groups, while the PIRS-1/IRS-1 ratio remained unchanged among the study groups. Our finding is consistent with previous studies by Pereira *et al.*, who reported that HTD enhanced insulin insensitivity with the involvement of the phosphorylation of PKC delta [20]. A previous study also demonstrated that free fatty acid-induced insulin resistance was associated with the suppression of protein kinase C delta (PKC δ) and downstream AKT [21]. Taken together, these data support the hypothesis that *trans*-fatty acids might induce more severe IR than HTD through reducing the expression of total IRS1, and suppressing the activation of phosphor- PKC δ Ser359 and phosphor- AKT Ser473.

Previous studies reported that IR was directly related with obesity and lipid metabolism dysfunction presented with increased serum TG and TC [22]. Intriguingly, no significant difference was observed between the ND and LTD groups, as well as between the HFD and HTD groups in terms of serum TC and TG levels post 12 and 20 weeks diet. This phenomenon could be attributed to several reasons: firstly, all mice in our study were the C57BL/6 background mice, which were not sensitive to high calories or *trans*-fatty acid-induced dyslipidemia [23,24]. Secondly, the regulation of *trans*-fatty acid on lipid transporter proteins might play a compensatory role to balance the negative effects of *trans*-fatty acids and fat overconsumption on lipid profiles [12]. Thirdly, most probably, more than 20 weeks’ time is needed to induce dyslipidemia in C57BL/6 mice by *trans*-fatty acid and high fat diets. Despite the similar lipid profiles between HFD and HTD mice, we observed more severe liver steatosis post *trans*-fatty acid diet. Asai A *et al.* [25] reported that liver steatosis could be more easily induced in C57BL/6 mice under the high-fat and high-carbohydrate diet than other background mice. This can partly illuminate the phenomenon whereby *trans*-fatty acid and HFD might induce significant liver steatosis without significantly affecting the lipid profiles.

Hepatic steatosis and insulin resistance are among the most prevalent metabolic disorders and tightly associated with obesity and type 2 diabetes mellitus (T2DM) [26]. Recent studies have reported that a high fat diet could induce insulin resistance and hepatic steatosis [18]. Our results demonstrated that *trans*-fatty acids could induce more severe hepatic steatosis in comparison with a high fat diet. To address the underlying mechanism, we examined DGAT1, ATGL and ACSL1 changes in the liver. DGAT1,diacyglycerol acy1 synthetase, is known to catalyze the conversion of diacylglycerol into TG [8]. ACS, an acyl CoA synthase, is known to promote the synthesis of acyl-CoAs [27]. Acy1-CoAs are important substrates for many synthesis pathways [28]. Joseph R. *et al.* [11] has reported that the ACSL1 expression level was associated with increased lipid loading as well as increased insulin sensitivity. ATGL, an adipose triglyceride lipase, participates actively in the hydrolysis of TG and could promote the lipid turnover to balance the lipid metabolism [29]. Excess lipid in the liver is stored in droplets as neutral TG, which is synthesized from fatty-CoA and diacylglycerol (DAG) in a reaction that is catalyzed by these proteins, then the body could compensate for overtaking fats [23]. Diacyglycerol acyl synthetase, lipoprotein lipase, adipose triglyceride lipase and acyl-CoA synthetase are all involved in the fatty acid uptake and lipid turn-over process. In the obesity and insulin resistance model, the expressions of these proteins were shown to be suppressed. These enzymes can work independently and affect each other [30]. In our study, HTD mainly suppressed the expression of ATGL, which might be responsible for dysfunctional lipid turnover in the liver, so the liver steatosis was worse in HTD mice. This suggested that *trans*-fatty acid might induce more severe liver steatosis than high fat diet, possibly through suppressing ATGL and subsequently promoting lipid accumulation in the liver. However, the effect of the activity of those enzymes can not be ignored.

In summary, consumption of a diet high in *trans*-fatty acids induces higher rates of obesity, IR and hepatic steatosis in male C57BL/6 mice. Possibly *trans*-fatty acid promotes lipid accumulation in the liver through suppressing the expression of ATGL, and *trans*-fatty acid might induce more severe IR by suppressing the IRS1 dependent insulin signal pathway. Obviously, our data provide substantial evidence for the importance of healthy food choices in terms of public health.

## 4. Materials and Methods

### 4.1. Animals and Diets

All animal procedures described in this study were approved by the Institutional Animal Care and Use Committee office of Fudan University and the treatment procedure was in compliance with the internationally recognized guidelines (Approval Number: 20160660C001). Eight-week-old male C57BL/6 mice were obtained from the Slack Laboratory Animal Company (Shanghai, China). Different diets as described below were started after two weeks of standard diet and continued for 20 weeks (*n* = 10 per group): ND, normal diet (25% kcal from fat) without Primex [31]; HFD, high fat diet (50% kcal from fat) without Primex; LTD, low *trans*-fatty acid diet (25% kcal from fat) with 97 g/kg Primex; HTD, high *trans*-fatty acid diet on the basis of high fat diet (50% kcal from fat). Table 1 show the detailed description of four diet regiments and all diets were bought from Research Diet Inc. (New York, NY, USA). The mice were housed in standard condition at 22 °C under a 12-h light/dark cycle with free access to diets and water.

### 4.2. Fat Mass and Sections of White Adipose Tissue

After the sacrifice, the white adipose tissue was collected from the abdominal subcutaneous and that was around the gonads. The brown fat was collected under the shoulder ministry skin. Then both white adipose tissue and brown fat was weighed. The fat mass in our study was the sum weight of white adipose tissue and brown fat.

### 4.3. Serum Lipid Profiles

Mice were fasted for 8 h and then blood samples were collected from angular vein using capillary glass tubes after anesthesia by isoflurane. After centrifugation at 3000 rpm, 15 min, plasma were inhaled into tubes [32]. Plasma triglyceride (TG), total cholesterol (TC) were detected by means of GPO-PAP method using commercial kits (Biosin Bio-Technology and Science Inc., Beijing, China).

### 4.4. Glucose Tolerance Test

Glucose tolerance test (GTT) was performed following 12-week and 20-week diet as described previously [33]. In brief, after fasting for 12 h, mice were injected intraperitoneally with 2 g/kg body weight of glucose. Then blood samples were drawn from the tail vein at baseline (0), 30, 60, 90, and 120 min after injection. Blood glucose was measure by an automatic glucometer (Life Scan Inc., London, UK).

### 4.5. Hematoxylin and Eosin (HE) Staining

The liver was arrested after anesthesia by isoflurane. For histological analysis, liver tissues were fixed in 10% neutral formaldehyde at room temperature for more than 24 h. The specimen was processed by graded alcohol, cleared in xylenes, and then embedded in paraffin. Serial sections were cut at 5 μm in the short axis, and stained with hematoxylin and eosin (HE) as described to analyze hepatocyte morphology and oil droplet [34]. Then we made a statistic about the vacuole that is oil droplet in the liver using the Image J software (Version 1.50, National Institutes of Health, Bethesda, MD, USA).

### 4.6. Oil Red O Staining

The tissue masses of liver were embedded in the Tissue-Tek compound and were frozen in the liquid nitrogen. The frozen liver was sectioned at 5-μm thickness with a Leica microtome (Leica Microsystems, Wetzlar, Germany) prior to fixation in 4% paraformaldehyde for 20 min. Following three washes with distilled water (5 min every time), the slides were placed in absolute propylene glycol for 5 min. Slides were then incubated in pre-warmed Oil Red O solution for 10 min in an oven at 60 °C [35]. Slides were rinsed twice with distilled water, mounted with aqueous mounting media and cover slipped. Lipid accumulation was digitalized using an Olympus BX-51 light microscope (Olympus America Inc., Melville, NY, USA) and measured with the Image J software.

### 4.7. Western Blot

Liver samples were quickly-frozen in liquid nitrogen and stored at −80 °C. Procedures of protein extraction and western blot were carried out as described previously [36]. Polyclonal goat antibody against DGAT1 (Sigma-Aldrich Inc., St. Louis, MO, USA), polyclonal mice antibody against monoclonal rabbit antibody against ATGL and polyclonal rabbit antibody against ACSL1 (Abcam Inc., Cambridge, MA, USA) and polyclonal rabbit antibodies against IRS1, Phospho-IRS1 (Ser302), PI3Knase (PI3K), Phospho-PI3Knase p85 (Tyr458)/p55 (Tyr199), AKT, Phospho-AKT(Ser473), PKCδ, Phospho-PKCδ (Ser359) (Cell Signaling Technology Inc., Danvers, MA, USA); Horseradish peroxidase conjugated secondary antibody (KANGCHEN Biotechnology, Nanjing, China) were examined by standard western blotting protocol. Respective proteins were analyzed with GAPDH serving as the loading control.

### 4.8. Statistical Analysis

Data were expressed as mean ± standard deviation (SD). Multi-group comparison was estimated by one-way analysis of variation (ANOVA) followed by a Bonferroni’s test for post hoc analysis. Analysis was accomplished with Graph Pad Prism 5.0 software (Graph Pad, San Diego, CA, USA). A *p* value < 0.05 is considered statistically significant.

## Figures and Tables

**Table 1:** Composition of normal diet (ND), high fat diet (HFD), *trans*-fatty acid diet (LTD) and high *trans*-fatty acid diet (HTD).

Ingredient	ND	HFD	LTD	HTD
Protein (%)	20	23	20	23
Carbohydrate (%)	60	39	60	39
Total fat (%)	12	28	12	28
Total Kcal (%)	100	100	100	100
Casein (g/kg)	200	200	200	200
DL-Methionine (g/kg)	3	3	3	3
Corn Starch (g/kg)	360	50	360	50
Maltodextrin 10 (g/kg)	50	125	50	125
Sucrose (g/kg)	203	161	203	161
Cellulose (g/kg)	50	50	50	50
Soybean Oil (g/kg)	25	25	25	25
Olive Oil (g/kg)	97	220	0	0
Primex (Vegetable shortening) (g/kg)	0	0	97	220
Mineral Mix (g/kg)	35	35	35	35
Vitamin Mix (g/kg)	10	10	10	10

Primex is an all-purpose creamy solid shortening made from a selected blend of partially hydrogenated soybean and palm oil stocks, rich in *trans*-fatty acids, with added dimethylpolysiloxane to control foaming [31].
